# Association Analysis of Markers Derived from Starch Biosynthesis Related Genes with Starch Physicochemical Properties in the USDA Rice Mini-Core Collection

**DOI:** 10.3389/fpls.2017.00424

**Published:** 2017-04-03

**Authors:** Kehu Li, Jinsong Bao, Harold Corke, Mei Sun

**Affiliations:** ^1^School of Biological Sciences, University of Hong KongHong Kong, China; ^2^Institute of Nuclear Agricultural Sciences, College of Agriculture and Biotechnology, Zhejiang UniversityHangzhou, China; ^3^Department of Food Science and Engineering, Shanghai Jiao Tong UniversityShanghai, China

**Keywords:** marker-trait association, rice, starch biosynthesis, starch physicochemical properties, USDA rice mini-core collection, *Waxy* gene haplotype

## Abstract

Rice eating and cooking quality is largely determined by starch physicochemical properties. The diverse accessions in the USDA rice mini-core collection (URMC) facilitate extensive association analysis of starch physicochemical properties with molecular markers specific to starch biosynthesis related genes. To identify significant trait-marker associations that can be utilized in rice breeding programs for improved starch quality, we conducted two association analyses between 26 molecular markers derived from starch biosynthesis related genes and 18 parameters measured of starch physicochemical properties in two sets of the mini-core accessions successfully grown in two environments in China. Many significant trait-marker associations (*P* < 0.001) were detected in both association analyses. Five markers of *Waxy* gene, including the (CT)_n_ repeats, the G/T SNP of intron 1, the 23 bp sequence duplication (InDel) of exon 2, the A/C SNP of exon 6, and the C/T SNP of exon 10, were found to be primarily associated with starch traits related to apparent amylose content (AAC), and two markers targeting the 4,329–4,330 bp GC/TT SNPs and 4,198 bp G/A SNP of *SSIIa* gene were mainly associated with traits related to gelatinization temperature (GT). Two new haplotypes were found in the mini-core collection based on the combinations of the 23 bp InDel and three SNPs (G/T of intron 1, A/C of exon 6, and C/T of exon 10) of *Waxy* gene. Furthermore, our analyses indicated that the (CT)_n_ polymorphisms of *Waxy* gene had a non-negligible effect on AAC related traits, as evidenced by significant variation in AAC related traits among rice accessions with the same *Waxy* SNPs but different (CT)_*n*_ repeats. As the five *Waxy* markers and the two *SSIIa* markers showed consistent major effects on starch quality traits across studies, these markers should have priority for utilization in marker-assisted breeding.

## Introduction

As the major component in rice grain, starch accounts for over 90% of endosperm weight, and thus the physicochemical properties of starch have foremost influence on rice eating and cooking quality. Two types of starch polymers exist in rice endosperm: amylose and amylopectin. Amylose is linear with few branches while amylopectin is highly branched. Amylose content and the fine structure of amylopectin are the main determinants of rice eating and cooking quality (Juliano, 1985; Bao et al., 2009; Syahariza et al., 2013; Kong et al., 2015). Apparent amylose content (AAC), gelatinization temperature (GT) and gel consistency had been established and still routinely used for grain quality evaluation (Juliano, 1985). Additional indicators have also been developed for more precise evaluation of grain quality, such as pasting viscosity profile, thermal, and retrogradation properties. All these starch physicochemical properties could be grouped as AAC-related traits and GT-related traits according to previous studies (Wang et al., 2007; Yang et al., 2014).

Starch biosynthesis in rice is affected by a concerted network of many enzymes. Generally, four classes of enzymes are involved in starch biosynthesis. They are adenosine diphosphate pyrophosphorylase (AGPase), starch synthase (SS), starch branching enzymes (SBEs), and starch de-branching enzymes (DBEs) (Martin and Smith, 1995; Smith et al., 1997). Among them, amylose biosynthesis is primarily controlled by granule bound starch synthase-I (insoluble starch synthase; He et al., 1999) while amylopectin production is a teamwork of SS (soluble starch synthase), SBEs and DBEs with distinct roles. SS catalyzes the transfer of the glucosyl moiety of ADP-glucose to the reducing end of pre-existing α-1,4 linked glucan chains; SBEs cleave α-1,4 chains and transfer them to C_6_ hydroxyls to form the branches α-1,6 chains. DBEs remove improperly branched α-1,6 chains within branched clusters (Nakamura, 2002; Nakamura et al., 2010).

The complexity of the process of rice starch biosynthesis is increased by the fact that those enzymes all have isoforms encoded by different genes. The *AGPase* has four large (*AGPL1-4*) and two small (*AGPS1, AGPS2*) catalytic subunits (Lee et al., 2007). A total of 10 isoforms in five types of starch synthase enzymes were reported: *GBSS* (*I, II*), *SSI, SSII* (*SSIIa, SSIIb, SSIIc*), *SSIII* (*SSIIIa* and *SSIIIb*), *SSIV* (*SSIVa* and *SSIVb*; Tatsuro and Tomio, 2004). Three isoforms of SBE: *SBEI, SBEII* (*SBEIIa, SBEIIb*; Yamanouchi and Nakamura, 1992) and two types of DBE: isoamylase and pullulanase have been reported (Nakamura et al., 1996; Fujita et al., 1999). The nucleotide sequence variation in these starch biosynthesis related genes can affect both amount and function of corresponding enzymes, and consequently the content and fine structure of amylose and amylopectin will be changed, leading to variation in starch physicochemical properties (Wang et al., 1995; Ayres et al., 1997; Larkin and Park, 2003; Umemoto and Aoki, 2005; Waters et al., 2006; Yu et al., 2011).

Association analysis is a powerful method to dissect the relationship between gene sequence polymorphisms and phenotypic variations (Thornsberry et al., 2001; Gupta et al., 2005). Many association analyses have been conducted on rice landraces and cultivars from different germplasm resources (see Table 1 in Zhang et al., 2016), but most of these studies are centered on agronomic traits in relation to yield, flowering time and disease resistance. Relatively few association analyses investigated starch physicochemical properties. Moreover, most of the studies were conducted on a limited number of starch traits using less diverse rice germplasm or on a limited number of molecular markers or markers that are not related to starch synthesis, which could make their findings unrepeatable. For example, Ayres et al. (1997) reported a very strong relationship between *Waxy* microsatellite alleles and AAC, but later studies confirmed this was largely due to the narrow genetic base in the employed samples (Bergman et al., 2000). As many markers derived from genes involved in starch biosynthesis are now available, it is necessary to investigate the utility of these markers with diverse rice germplasm for a better understanding of the genetic basis of starch quality traits.

The USDA rice mini-core collection (URMC) consists of 217 accessions that were selected to represent over 18,000 accessions in the USDA global gene bank of rice (Agrama et al., 2009). It has been used in QTL mapping for genes associated with grain yield and other agronomic traits, disease resistance, or protein concentration (Li et al., 2011, 2012; Jia et al., 2012; Bryant et al., 2013). In this study, we conducted two association analyses to investigate the relationships between 26 molecular markers derived from 18 starch biosynthesis related genes and 18 parameters of starch physicochemical properties measured for diverse URMC accessions successfully grown in two environments in China. Our aim was to compare the importance of these molecular markers in determining starch traits and to find the most efficient markers or marker combinations/haplotypes that can be used in developing rice cultivars with improved cooking and eating quality via marker-assisted breeding.

## Materials and methods

### Plant materials

Samples of 217 accessions from the USDA rice mini-core collection were provided by U.S. Department of Agriculture—Agricultural Research Service (USDA-ARS). The accessions were grown in Hainan (18° N) from December 2013 to April 2014, and in Hangzhou (18° N) from June to October in 2014, in a randomized block design with two replications within each environment. In each replicate, two rows and six plants per row for each accession were planted at a spacing of 20 cm between and within rows. However, only 160 accessions in Hainan and 155 in Hangzhou produced enough seeds for measurement of starch traits.

### Measurement of starch physicochemical properties

The rice seeds harvested from the two environments Hainan and Hangzhou were measured for 18 parameters of starch physicochemical properties as described in Li et al. (2017). The AAC was determined by a colorimetric method. Gel texture properties including hardness (HD), adhesiveness (ADH), cohesiveness (COH) were measured by TA-XT2i Texture Analyzer (Texture Technologies Corp., Scarsdale, NY). RVA pasting profile was determined by a Rapid Visco Analyzer (RVA, Model 3-D, Newport Scientific, Warriewood, Australia), and the measured parameters include peak viscosity (PV), hot paste viscosity (HPV), cool paste viscosity (CPV), peak time (Ptime), pasting temperature (PT) and three derivative parameters: breakdown (*BD* = PV-HPV), setback (*SB* = CPV-PV), and consistency (*CS* = CPV-HPV). Thermal properties were measured using a DSC 2,920 thermal analyzer (TA Instruments, Newcastle, DE, USA), and the measured parameters include onset temperature (T_*o*_), peak temperature (T_*p*_), conclusion temperature (T_*c*_), enthalpy of gelatinization (ΔHg), width at half peak height (ΔT_1/2_), and percentage of retrogradation (R%). All these parameters were measured in duplicate, and the mean values of the 18 measured parameters were used for association analysis.

Wide variation was observed in all 18 parameters measured of starch physicochemical properties in both sets of rice samples successfully grown in Hainan and Hangzhou (Li et al., 2017; Supplementary Table 1). For example, AAC ranged from 1.1 to 29.4% and averaged 20.6%, with CV of 35.44% in the 160 accessions harvested from Hainan. A similarly high level of variation in AAC was shown in the 155 accessions from Hangzhou. High genetic diversity was also observed in other parameters of starch physicochemical properties, such as gelatinization temperature (T_*p*_) which ranged from 66.7 to 81.0°C and from 66.1 to 82.3°C in the two sets of samples respectively. These parameters were used in the present association analysis to identify marker-trait relationships.

### DNA isolation and genotyping

Whole genomic DNA was extracted from five seedlings of each accession using the CTAB method of Doyle (1991).

Twenty-five molecular markers derived from 18 starch biosynthesis related genes were used for genotyping all 217 rice accessions for this study (Supplementary Table 2).

PCR reaction was performed in a 10 μl reaction mixture containing 20 ng of template DNA, 1X PCR buffer, 2 mM MgCl_2_, 0.2 mM dNTPs, 0.2 μM of each primer and 1 unit of Taq DNA polymerase. All amplifications were performed on a PTC-100 thermal cycler (MJ Research, Inc.) under following conditions: 5 min at 94°C, followed by 45 s at 94°C, 60 s at T_A_ (Supplementary Table 2), 60 s at 72°C for 35 cycles, and 7 min at 72°C for a final extension.

The PCR products of InDel and STS markers were either resolved on 2.0% agarose gel containing 0.05 μl/mL gel red in 1X TBE buffer and visualized using a gel documentation system, or separated on 8% polyacrylamide denaturing gels and visualized by silver staining (Bassam et al., 1991).

PCR products of SNP markers were digested with restriction endonucleases (New England BioLabs; Supplementary Table 2) according to manufacturer's instructions. The digests were separated on 8% polyacrylamide gel and visualized by silver staining.

### Statistical analysis

A total of 26 molecular markers were used in the association analysis. In addition to the 25 marker loci we genotyped for the 217 URMC accessions, the (CT)_n_ polymorphism of marker RM190 of *Waxy* gene reported in Li et al. (2010) was included in the final data analysis.

Data analysis was performed using the SAS system for windows version 8 (SAS Institute Inc., Cary, NC, USA). The Student-Newman-Keuls test was conducted for comparison of means at *P* < 0.05, and PROC GLM was used for analysis of variance determination.

PowerMarker (Version 3.25) was used to calculate the polymorphism information content (PIC) for the 26 markers and Nei's distance (Nei et al., 1983) between rice accessions. Based on Nei's distance, UPGMA was performed and the tree was viewed in MEGA 7.0.

### Association analysis

Using the same set of 26 molecular markers and the mean values of 18 measured parameters of starch traits, we conducted two separate association analyses for the 160 rice accessions grown in Hainan and 155 accessions grown in Hangzhou, respectively, using the mixed linear model (MLM) of TASSEL (Version 2.1) which takes both population structure (*Q*) and kinship (*K*) into account, and rare alleles (frequency <5%) were removed before association analysis. We estimated the parameters of *Q* and *K* based on 155 SSR markers reported in Li et al. (2010). The detection of marker-trait association was determined by the *p*-value (marker) (*p* < 0.01).

## Results

### Allelic diversity at the marker loci from 18 starch biosynthesis related genes

All the 26 gene specific markers were polymorphic in the 217 mini-core accessions, with polymorphic information content (PIC) ranging from 0.09 (marker *SSIVb-*Indel) to 0.85 (marker RM190; Table 1).

**Table 1:** **Polymorphism information content (PIC) of 26 markers derived from 18 starch biosynthesis related genes in rice accessions of different ancestry groups**.

	**Total *n* = 217**		**TRJ^1^*n* = 39**		**TEJ *n* = 32**		**IND *n* = 72**		**AUS *n* = 39**		**Admix *n* = 17**		**ARO *n* = 6**		**WD *n* = 12**	
	**No. of alleles**	**PIC**	**No. of alleles**	**PIC**	**No. of alleles**	**PIC**	**No. of alleles**	**PIC**	**No. of alleles**	**PIC**	**No. of alleles**	**PIC**	**No. of alleles**	**PIC**	**No. of alleles**	**PIC**
Waxy-intron 1	2	0.33	2	0.27	2	0.37	2	0.31	2	0.32	2	0.33	2	0.24	2	0.24
RM190	15	0.85	5	0.52	4	0.55	8	0.63	9	0.77	8	0.81	2	0.27	5	0.72
Waxy-exon 2	2	0.13	1	0.00	2	0.16	2	0.14	2	0.20	2	0.10	1	0.00	2	0.14
Waxy-exon 6	2	0.31	2	0.05	2	0.35	2	0.14	2	0.31	2	0.30	2	0.24	1	0.00
Waxy-exon 10	2	0.25	2	0.05	2	0.11	2	0.37	2	0.09	2	0.10	1	0.00	2	0.14
SSIIa-4329,4330	2	0.24	2	0.05	2	0.32	2	0.28	2	0.23	2	0.25	2	0.24	2	0.14
SSIIa-4198	2	0.14	1	0.00	2	0.37	2	0.03	1	0.00	2	0.10	1	0.00	2	0.14
SBEIIb (SBE3)	2	0.35	2	0.17	2	0.19	2	0.34	2	0.27	2	0.30	2	0.35	2	0.24
ISA3	2	0.37	2	0.23	2	0.06	2	0.08	1	0.00	2	0.25	2	0.24	2	0.24
ISA1	2	0.37	2	0.27	2	0.11	2	0.28	2	0.09	2	0.35	2	0.24	2	0.30
SSIVb	2	0.37	2	0.17	1	0.00	2	0.10	1	0.00	2	0.30	1	0.00	2	0.15
SSIVb-Indel	2	0.09	2	0.13	2	0.06	1	0.00	2	0.20	2	0.19	1	0.00	1	0.00
SSIIb-Indel	2	0.37	2	0.05	2	0.06	2	0.08	2	0.05	2	0.35	1	0.00	2	0.14
PUL-Indel	2	0.26	2	0.23	2	0.36	2	0.14	2	0.09	2	0.33	2	0.24	1	0.00
SSIIc (SSII-1)	2	0.37	2	0.05	1	0.00	2	0.05	2	0.17	2	0.30	1	0.00	2	0.15
SSIIIb (SSIII-1)	2	0.19	1	0.00	2	0.38	2	0.05	2	0.20	2	0.25	2	0.24	1	0.00
GBSSII	2	0.37	2	0.37	2	0.37	2	0.19	2	0.09	2	0.37	1	0.00	2	0.24
BEI (SBE1)	2	0.21	2	0.05	2	0.31	2	0.05	2	0.05	2	0.19	1	0.00	2	0.14
BEIIa (SBE4)	2	0.37	2	0.31	2	0.06	2	0.10	2	0.05	2	0.30	2	0.24	2	0.15
BEIIb (SBE3)	2	0.35	2	0.23	2	0.11	2	0.37	2	0.29	2	0.37	2	0.24	2	0.24
AGPL1	2	0.28	2	0.27	2	0.19	2	0.35	2	0.29	2	0.10	2	0.24	1	0.00
AGPS2-2	2	0.30	2	0.05	2	0.29	2	0.08	2	0.27	2	0.35	1	0.00	2	0.30
AGPL4-1	2	0.37	2	0.35	1	0.00	2	0.08	2	0.05	2	0.35	1	0.00	2	0.15
AGPS2-1	2	0.37	2	0.32	2	0.06	2	0.25	2	0.05	2	0.30	1	0.00	2	0.14
AGPS1	2	0.36	2	0.05	2	0.19	2	0.13	2	0.27	2	0.33	2	0.38	2	0.19
AGPL3	2	0.37	2	0.17	1	0.00	2	0.26	1	0.00	2	0.33	1	0.00	2	0.35
AGPS2-3	2	0.36	2	0.36	2	0.06	2	0.25	1	0.00	2	0.30	1	0.00	1	0.00

*TRJ, tropical japonica; TEJ, temperate japonica; IND, indica; AUS, aus; ARO, aromatic; WD, wild rice; Admix accessions with mixed ancestry*.

Following Li et al. (2010), the 217 accessions in the mini-core were divided into seven ancestry groups, and the PIC of the 26 markers in each group was calculated in the present study (Table 1). The “Admix” group, which refers to the rice accessions having the mixed ancestry (such as the TEJ-TRJ mixture, and the IND-AUS mixture), had two or more alleles at each of the 26 marker loci whereas allele fixation occurred at some of the loci in the other six groups. Allele fixation occurred at 14 marker loci in the ARO group, likely due to a small number of accessions (*n* = 6) belonging to the group. In contrast, only six loci were fixed in the wild rice (the WD group, *n* = 12). Compared to the ancestry groups TEJ, IND, and AUS, the TRJ group showed much lower PIC for four markers: 23 bp InDel of exon 2, A/C SNP of exon 6, and C/T SNP of exon 10 in *Waxy* gene, and the GC/TT SNPs in *SSIIa*.

**Figure 1:**
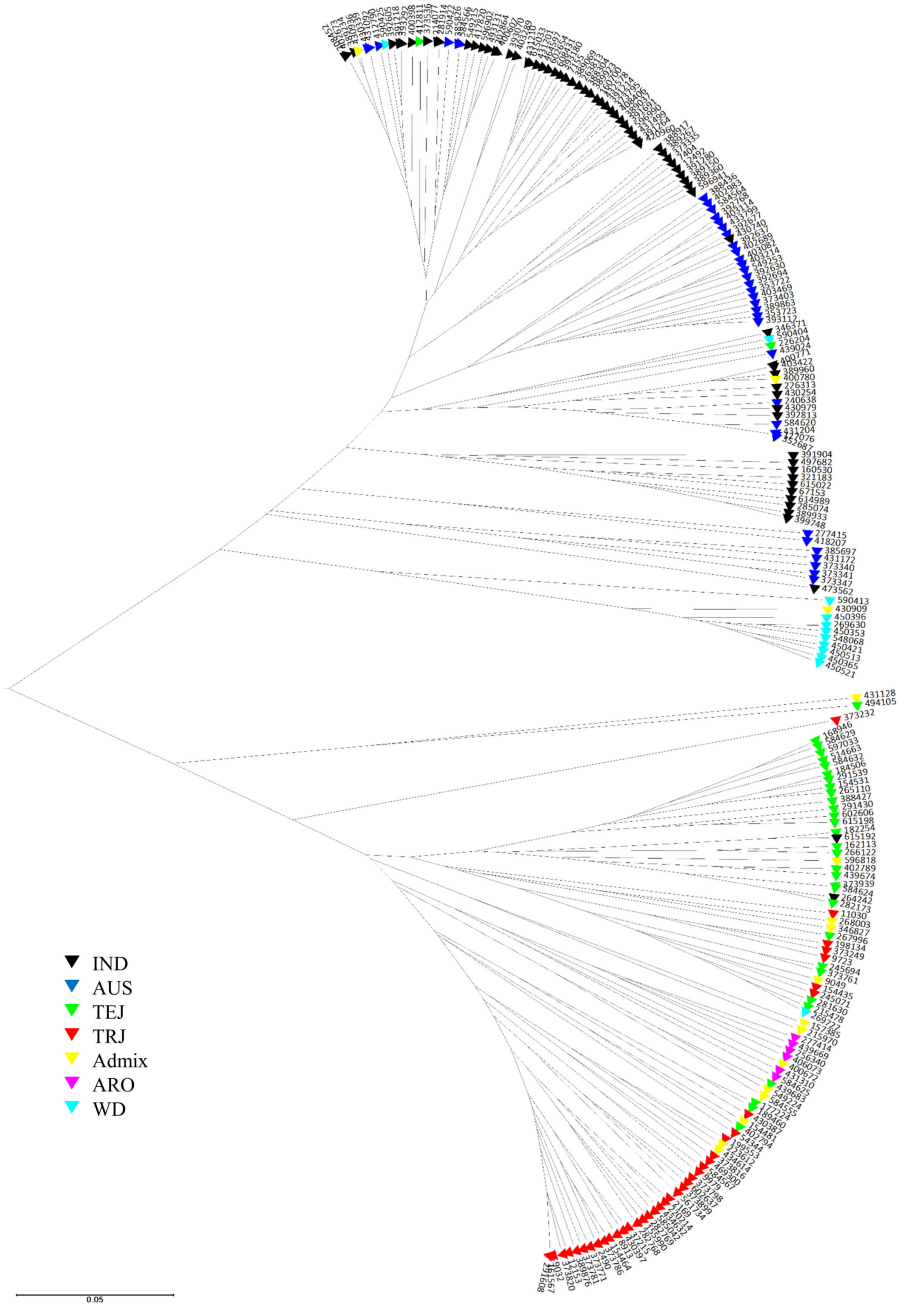
**UPGMA tree of 217 access in USDA rice mini-core collection based on Nei's genetic distance calculated from 26 markers derived from 18 starch biosynthesis genes**. The seven ancestry groups include IND *indica*, AUS *aus*, TEJ *temperate japonica*, TRJ *tropical japonica*, ARO *aromatic*, WD *wild rice*, and Admix *accessions with mixed ancestry* (classified according to Li et al., 2010).

UPGMA analysis based on Nei's genetic distance was used to visualize genetic relationships among the 217 accessions (Figure 1). Two major clusters were observed, one consisting of nearly all accessions of ancestry groups AUS, IND, and WD, and the other consisting of nearly all accessions of ancestry groups TEJ, TRJ, and ARO. Within each of the two major clusters, however, the accessions of the same ancestry group could be placed into several subgroups. As it might be expected, the accessions of Admix group spread into many subgroups both within and between the two major clusters.

### Association analysis

Results of the two marker-trait association analyses for the 160 rice accessions grown in Hainan and 155 accessions grown in Hangzhou were shown in Table 2. The common markers detected in both association analyses were all from *Waxy* gene and *SSIIa* gene. The *Waxy*-exon 2 marker was consistently detected as having association with nearly all parameters of starch physicochemical properties (except for thermal properties). The common markers detected for the parameters of pasting viscosity (PV, HPV, CPV, SB, BD, and Ptime) were all from *Waxy* gene. For parameters of thermal property, such as T_o_, T_p_, and T_c_, the common markers detected were all from *SSIIa* gene. For HD and ADH of gel texture and AAC, markers from both *Waxy* gene and *SSIIa* gene were detected. Interestingly, *Waxy*-exon 10 marker was found to have association with only HPV and CPV (*p* < 0.0001) in both analyses.

**Table 2:** **Marker-trait associations [*p*-Marker < 0.01; mixed linear model (*Q* + *K*)] detected in two environments Hainan and Hangzhou**.

**Trait**	**Hainan**		**Hangzhou**		***Waxy*** **background**				***SSIIa*** **background**			
					**Hainan**		**Hangzhou**		**Hainan**		**Hangzhou**	
	**Marker**	**p_Marker**	**Marker**	**p_Marker**	**Marker**	**p_Marker**	**Marker**	**p_Marker**	**Marker**	**p_Marker**	**Marker**	**p_Marker**
*AAC*									Waxy-Exon 2	2.19 × 10^−19^	Waxy-Exon 2	5.58 × 10^−7^
AAC	Waxy-Exon 2	1.08 × 10^−21^	Waxy-Exon 2	1.06 × 10^−8^	SSIIa GC/TT	2.50 × 10^−3^					Waxy-Exon 6	5.00 × 10^−3^
	RM190	2.35 × 10^−8^	RM190	8.98 × 10^−4^	SSIIIb AG/AGAG	5.60 × 10^−3^						
	SSIIa GC/TT	6.38 × 10^−7^	SSIIa GC/TT	2.01 × 10^−4^								
	Waxy-intron 1	2.32 × 10^−4^	Waxy-Exon 6	1.00 × 10^−3^								
	Waxy-Exon 6	6.50 × 10^−3^	Waxy-intron 1	6.10 × 10^−3^					RM190	6.50 × 10^−7^		
**GEL TEXTURE**												
HD	RM190	5.14 × 10^−13^	SSIIa GC/TT	2.56 × 10^−5^			SSIIa GC/TT	6.80 × 10^−−^	RM190	1.76 × 10^−11^	Isa3 A/G	2.10 × 10^−3^
	Waxy-Exon 2	9.43 × 10^−12^	Waxy-Exon 2	1.39 × 10^−4^					Waxy-Exon 2	4.40 × 10^−10^	Waxy-Exon 2	3.60 × 10^−3^
	Waxy-intron 1	5.11 × 10^−4^	RM190	2.16 × 10^−4^					Waxy-Exon 10	4.12 × 10^−4^	SSIIc G/T	3.90 × 10^−3^
	Waxy-Exon 10	3.11 × 10^−4^	Waxy-intron 1	5.70 × 10^−4^							RM190	6.20 × 10^−3^
	SSIIa GC/TT	1.50 × 10^−4^	Waxy-Exon 6	9.20 × 10^−3^								
			Isa3 A/G	6.30 × 10^−3^								
ADH	RM190	2.78 × 10^−13^	Waxy-Exon 2	8.73 × 10^−5^	SSIIIb AG/AGAG	4.00 × 10^−3^	L4^−^1	9.90 × 10^−3^	RM190	6.03 × 10^−12^	SSIIc G/T	1.30 × 10^−3^
	Waxy−Exon 2	2.12 × 10^−12^	SSIIa GC/TT	4.35 × 10^−4^	SBEIIa C/G	6.20 × 10^−3^			Waxy-Exon 2	8.66 × 10^−11^	Waxy−Exon 2	1.60 × 10^−3^
	SSIIa GC/TT	7.46 × 10^−4^	RM190	5.77 × 10^−4^					Waxy-Exon 10	4.40 × 10^−3^	RM190	5.90 × 10^−3^
	Waxy-Exon 10	3.31 × 10^−3^	SSIIc G/T	3.80 × 10^−3^							Isa3 A/G	6.20 × 10^−3^
	Waxy-intron 1	5.50 × 10^−3^										
COH	Waxy-Exon 2	3.85 × 10^−8^	Waxy-Exon 2	7.72 × 10^−6^			SSIIa GC/TT	7.40 × 10^−3^	Waxy-Exon 2	2.63 × 10^−7^	Waxy-Exon 2	9.76 × 10^−5^
	SSIIc G/T	5.20 × 10^−3^	SSIIa GC/TT	5.70 × 10^−3^					SSIIc G/T	2.07 × 10^−4^	RM190	4.60 × 10^−3^
			RM190	7.40 × 10^−3^					AGPS2-2	4.80 × 10^−3^		
**PASTING VISCOSITY**												
PV	Waxy-Exon 2	1.13 × 10^−5^	Waxy-Exon 2	9.00 × 10^−3^					Waxy-Exon 2	8.72 × 10^−7^	Waxy-Exon 2	5.10 × 10^−3^
	Waxy-Exon 10	4.38 × 10^−4^							Waxy-Exon 10	2.76 × 10^−4^		
HPV	Waxy-Exon 10	1.64 × 10^−7^	Waxy-Exon 10	4.00 × 10^−6^	SSIIa GC/TT	1.60 × 10^−3^	SSIIIb AG/AGAG	5.54 × 10^−4^	Waxy-Exon 10	4.07 × 10^−8^	Waxy-Exon 10	1.41 × 10^−6^
	Waxy-Exon 2	2.25 × 10^−5^	SSIIIb AG/AGAG	2.70 × 10^−3^	SBEIIb A/G	5.20 × 10^−3^	SSIIb Indel	5.50 × 10^−3^	Waxy-Exon 2	1.28 × 10^−6^	Waxy-Exon 2	2.50 × 10^−3^
	RM190	1.53 × 10^−4^	Waxy-Exon 2	8.50 × 10^−3^			SBEIIb A/G	7.70 × 10^−3^	RM190	7.39 × 10^−5^	SSIIIb AG/AGAG	8.40 × 10^−3^
									Waxy-intron 1	8.70 × 10^−7^		
CPV	Waxy-Exon 2	9.94 × 10^−18^	Waxy-Exon 2	1.75 × 10^−10^	SBEIIb A/G	9.20 × 10^−3^	SSIIIb AG/AGAG	3.50 × 10^−4^	Waxy-Exon 2	1.73 × 10^−17^	Waxy-Exon 2	5.08 × 10^−7^
	RM190	1.14 × 10^−8^	Waxy-Exon 10	2.70 × 10^−5^			SSIIb Indel	3.60 × 10^−3^	RM190	2.02 × 10^−8^	Waxy−Exon 10	3.58 × 10^−5^
	Waxy-Exon 10	1.23 × 10^−7^	SSIIIb AG/AGAG	8.60 × 10^−3^			SSIIb C/G	8.10 × 10^−3^	Waxy-Exon 10	1.94 × 10^−7^	SSIIIb AG/AGAG	6.50 × 10^−3^
	Waxy-intron 1	3.21 × 10^−5^	SSIIb Indel	9.20 × 10^−3^					Waxy-intron 1	7.32 × 10^−5^	SSIIb Indel	8.10 × 10^−3^
CS	Waxy-Exon 2	2.63 × 10^−21^	Waxy-Exon 2	4.09 × 10^−8^					Waxy-Exon 2	1.67 × 10^−19^	Waxy-Exon 2	7.45 × 10^−7^
	RM190	2.98 × 10^−8^	Waxy-intron 1	2.60 × 10^−3^					RM190	2.39 × 10^−7^		
	Waxy-intron 1	3.47 × 10^−6^	Waxy-Exon 6	4.20 × 10^−3^					Waxy-intron 1	7.85 × 10^−5^		
	Waxy-Exon 10	9.56 × 10^−5^	SSIIa GC/TT	5.90 × 10^−3^					Waxy-Exon 10	1.40 × 10^−4^		
	SSIIa GC/TT	1.30 × 10^−3^										
	Waxy-Exon 6	9.50 × 10^−3^										
SB	Waxy-intron 1	2.89 × 10^−6^	Waxy-intron 1	7.40 × 10^−3^	SSIIIb AG/AGAG	3.00 × 10^−3^	SSIIIb AG/AGAG	9.10 × 10^−3^	Waxy-intron 1	1.28 × 10^−5^	Waxy-intron 1	7.50 × 10^−3^
	Waxy-Exon 2	1.08 × 10^−5^	Waxy-Exon 2	4.50 × 10^−3^					Waxy-Exon 2	4.03 × 10^−5^		
	RM190	1.51 × 10^−5^										
	SSIIa GC/TT	7.00 × 10^−3^							RM190	7.28 × 10^−5^		
BD					SSIIa G/A	6.10 × 10^−3^			Waxy-intron 1	5.00 × 10^−3^		
Ptime	Waxy-Exon 2	6.46 × 10^−35^	Waxy-Exon 2	1.10 × 10^−17^	SBEIIb A/G	3.60 × 10^−3^	SSIIIb AG/AGAG	4.20 × 10^−3^	Waxy-Exon 2	4.37 × 10^−36^	Waxy-Exon 2	1.30 × 10^−18^
	RM190	5.66 × 10^−4^			SSIIb-Indel	3.70 × 10^−3^			RM190	9.87 × 10^−4^		
	Waxy-Exon 6	6.33 × 10^−4^			SSIIa G/A	3.70 × 10^−3^			Waxy-Exon 6	1.00 × 10^−3^		
	Waxy-Exon 10	8.90 × 10^−3^										
	Waxy-intron 1	7.40 × 10^−3^										
PT	Waxy-Exon 2	6.93 × 10^−10^	SSIIa GC/TT	2.41 × 10^−9^	SSIIa GC/TT	4.31 × 10^−7^	SSIIa GC/TT	6.75 × 10^−8^	Waxy-Exon 2	2.37 × 10^−7^	Waxy-Exon 2	4.71 × 10^−4^
	SSIIa GC/TT	1.20 × 10^−9^	Waxy-Exon 2	9.36 × 10^−6^					Waxy-Exon 6	7.12 × 10^−4^		
	Waxy-Exon 6	4.11 × 10^−4^							SSIIc G/T	3.00 × 10^−3^		
									ISA3 A/G	6.70 × 10^−3^		
**THERMAL PROPERTIES**												
T_*o*_	SSIIa GC/TT	5.46 × 10^−11^	SSIIa GC/TT	5.52 × 10^−14^	SSIIa GC/TT	1.93 × 10^−10^	SSIIa GC/TT	2.55 × 10^−13^	SSIIc G/T	3.86 × 10^−5^	Waxy-intron 1	5.20 × 10^−3^
	SSIIa G/A	9.73 × 10^−6^	SSIIa G/A	3.10 × 10^−3^	SSIIa G/A	2.36 × 10^−5^	SSIIa G/A	6.30 × 10^−3^	Waxy−intron 1	7.19 × 10^−4^		
	Waxy-Exon 2	1.20 × 10^−3^							SBEIIb-STS	3.57 × 10^−4^		
									ISA3 A/G	2.97 × 10^−4^		
									AGPS2-2	1.20 × 10^−3^		
									SSIVb G/A	9.10 × 10^−3^		
T_*p*_	SSIIa GC/TT	2.19 × 10^−12^	SSIIa GC/TT	6.58 × 10^−14^	SSIIa GC/TT	1.57 × 10^−12^	SSIIa GC/TT	1.41 × 10^−13^	SSIIc G/T	3.77 × 10^−5^	Waxy-intron 1	6.10 × 10^−3^
	SSIIa G/A	4.17 × 10^−4^	SSIIa G/A	4.17 × 10^−4^	SSIIa G/A	6.69 × 10^−4^			SBE1-STS	1.06 × 10^−4^		
									ISA3 A/G	2.95 × 10^−4^		
									Waxy-intron 1	3.86 × 10^−4^		
									SSIIb-Indel	7.70 × 10^−3^		
									AGPS2-2	5.30 × 10^−3^		
T_*c*_	SSIIa GC/TT	9.32 × 10^−6^	SSIIa GC/TT	5.28 × 10^−10^	SSIIa GC/TT	3.02 × 10^−6^	SSIIa GC/TT	9.47 × 10^−11^	SSIIc G/T	2.60 × 10^−3^		
	SSIIa G/A	7.15 × 10^−4^			SSIIa G/A	9.40 × 10^−4^			SSIIb-Indel	2.70 × 10^−3^		
									Waxy-intron 1	9.00 × 10^−3^		
									ISA3 A/G	9.80 × 10^−3^		
ΔT_1/2_	Waxy-Exon 2	1.16 × 10^−4^	SSIIa GC/TT	1.08 × 10^−7^	AGPS1	3.90 × 10^−3^	SSIIa GC/TT	4.65 × 10^−7^	Waxy-Exon 2	1.70 × 10^−3^	SSIVb G/A	1.20 × 10^−3^
			SSIIa G/A	2.00 × 10^−4^			SSIIIb AG/AGAG	9.30 × 10^−4^				
			RM190	1.20 × 10^−3^			SSIIa G/A	9.68 × 10^−4^				
			SSIIIb AG/AGAG	1.90 × 10^−3^			BEI−STS	7.90 × 10^−3^				
			BEI-STS	7.00 × 10^−3^			SSIVb A/G	8.70 × 10^−3^				
ΔHg							SSIVb A/G	9.50 × 10^−3^				
R	Waxy-Exon 2	4.90 × 10^−3^	SSIIb Indel	9.70 × 10^−3^							SSIIb Indel	3.90 × 10^−3^

The major difference between the two analyses was in *P*-values (p_Marker in Table 2). For example, association between *Waxy*-exon 2 and AAC was detected with *P*-value of 1.08 × 10^−21^ in the Hainan samples, but a much lower *P* level (1.06 × 10^−8^) was found in the Hangzhou samples for the same association. There were also some other differences between the two analyses. For example, the *Waxy*-exon 10 and HD association (3.11 × 10^−4^) was detected only in the Hainan samples, suggesting a sampling effect in the marker-trait association analysis.

In order to detect other markers that could differentiate trait variation under the same *Waxy* or *SSIIa* marker background, further association analyses were conducted with the five *Waxy* markers [(CT)_n_ repeats, G/T SNP in intron 1, 23 bp InDel in exon 2, A/C SNP in exon 6, C/T SNP in exon 10] and two *SSIIa* markers (G/A SNP at 4198 bp and GC/TT SNPs at 4,329–4,330 bp) set as covariates, respectively (Table 2). Under the *Waxy* background, associations were found between markers from *SSIIa* gene and parameters PT, T_o_, T_p_, and T_c_ in both analyses. In contrast, few common markers were found for other starch traits. Only *SBEIIb* A/G and *SSIIIb* AG/AGAG were found for HPV and SB respectively in both analyses. Under the *SSIIa* background, the *Waxy*-intron 1 marker was detected only for two parameters T_*o*_ and T_*p*_, but the associations of *Waxy*-exon 2 marker were detected with many parameters, such as AAC, HD, ADH, COH, PV, HPV, CPV, CS, Ptime, and PT. In addition, the associations of *Waxy*-exon 10 with HPV and CPV were detected in both analyses.

### *Waxy* haplotypes in the rice accessions grown in hainan

**Figure 2:**
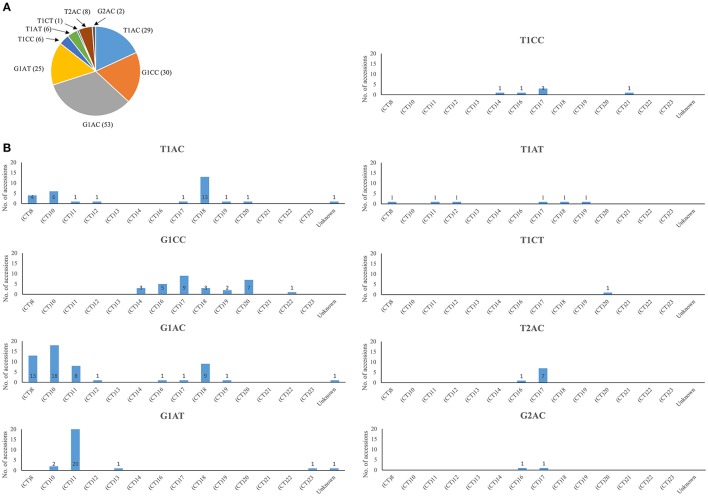
***Waxy*** **haplotype frequencies in rice accessions used in the present study**. **(A)** *Waxy* SNP combinations (including the 23 bp InDel in exon 2) and numbers of associated accessions. **(B)** Association of (CT)_n_ classes with SNP combinations.

A total of nine *Waxy* haplotypes were identified in the 160 accessions grown in Hainan based on the allelic combinations of the three SNPs and the 23 bp InDel in *Waxy* gene (Figure 2A). There were 137 accessions falling into T1AC, G1CC, G1AC, and G1AT haplotype groups. The G1AC group consisted of most accessions (53 accessions) while the other three haplotypes had similar numbers of accessions (from 25 to 30 accessions). Two haplotypes (T1AT and T1CT) among the nine had not been discovered before. However, these haplotypes may be rare in nature, as they could only be found in a small number of accessions: six accessions with the T1AT haplotype, and only one accession with the T1CT haplotype (Supplementary Table 3).

A total of 14 *Waxy* (CT)_*n*_ microsatellite alleles were identified (Figure 2B). The top five most frequent alleles were (CT)_11_, (CT)_10_, (CT)_18_, (CT)_17_, and (CT)_8_,found in 30, 26, 26, 23, and 18 accessions respectively. The other nine alleles were each found in <10 accessions. Consistent with previous reports Ayres et al., 1997; Bao et al., 2006a; Chen et al., 2008), short repeats of (CT)_n_ = 8, 10, 11, and long repeats of (CT)_*n*_ = 17, 18, 20 were found to be more frequent in the rice accessions.

The associations of SNP combinations (including the 23 bp Indel in exon 2) and the (CT)_n_ classes of *Waxy* gene were shown in Figure 2B. The T2AC and G2AC haplotypes (harboring a 23 bp duplication in exon 2) were both associated with (CT)_16_ or (CT)_17_. Most rice (20 out of 25) with the G1AT haplotype were associated with the (CT)_11_ allele. The G1AC haplotype was mainly associated with short (CT)_n_ repeats of 8, 10, 11, and long (CT)_n_ repeat of 18; The T1AC haplotype was mainly associated with short (CT)_n_ repeats of 8, 10, and long (CT)_n_ repeat of 18. Rice accessions with G1CC haplotype were associated with (CT)_n_ groups of 14, 16, 17, 18, 19, and 20. Other rare haplotypes were found in several (CT)_n_ groups.

### Means and ranges of AAC related traits in rice accessions with different *Waxy* haplotypes

The accessions with haplotypes T2AC and G2AC had significant lower mean AAC (Table 3). The G1AT and G1AC groups all had a mean AAC higher than 23% while the mean AAC of T1AC and G1AC were all <23% but higher than 18%. Three accessions with T1AT haplotype were associated with short (CT)_n_ of 8, 11, 12 repeats, and they had AAC at 26.6%, 26.0% and 24.3% respectively.

Table 3**Mean and range of AAC related traits in rice accessions with different Waxy SNP combinations**.

**Table:** 

**Parameters**	**G1AC^1^ (*n* = 53)**		**G1AT (*n* = 25)**		**G1CC (*n* = 30)**		**T1AC (*n* = 28)**	
	**Mean ± SD**	**Range**	**Mean ± SD**	**Range**	**Mean ± SD**	**Range**	**Mean ± SD**	**Range**
AAC (%)	23.5^a^ ± 5.6	4.2–28.7	25.0^a^ ± 2.0	20.6–29.4	19.7^b^ ± 4.1	3.5–28.1	18.8^b^ ± 7.6	8.4–28.5
HD (g)	51.1^b^ ± 16.5	16.5–6.6	63.9^a^ ± 12.0	43.5–89.7	36.8^c^ ± 11.9	9.7–65.6	36.3^c^ ± 22.6	12.3–78.9
ADH (g.s)	−76.0^b^ ± 22.6	−106.9 to −11.2	−84.5^b^ ± 14.8	−113.4 to −59.4	−48.5^a^ ± 16.4	−92.3 to −12.8	−57.4^a^ ± 34.8	−109.9 to −19.9
COH	0.6^a^ ± 0.0	0.5–0.7	0.6^b^ ± 0.0	0.5–0.6	0.6^a^ ± 0.0	0.5–0.6	0.6^a^ ± 0.0	0.5–0.6
PV (RVU)	286.1^c^ ± 50.4	212.0–453.2	356.6^a^ ± 26.5	295.7–411.1	319.2^b^ ± 30.0	262.5–374.7	333.3^ab^ ± 76.5	213.8–450.3
HPV (RVU)	163.6^c^ ± 28.2	118.3–242.0	222.9^a^ ± 26.5	157.5–286.5	180.6^b^ ± 20.6	138.3–223.9	179.9^b^ ± 36.1	109.4–253.5
CPV (RVU)	336.9^b^ ± 34.8	225.9–416.1	432.5^a^ ± 47.2	297.6–525.4	335.5^b^ ± 37.2	208.5–403.3	326.2^b^ ± 36.3	263.5–396.2
CS (RVU)	173.3^b^ ± 26.9	67.7–214.2	209.6^a^ ± 25.7	140.1–284.6	155.0^c^ ± 27.6	53.0–199.8	146.3^c^ ± 35.1	86.5–209.1
SB (RVU)	50.8^a^ ± 49.3	−113.2–128.5	75.9^a^ ± 38.4	−18.0 to 133.2	16.3^b^ ± 47.3	−66.5 to 121.8	−7.1^b^ ± 81.6	−148.6 to 100.6
BD (RVU)	122.5^b^ ± 33.8	64.9–252.4	133.7^ab^ ± 21.2	92.3–167.2	138.7^ab^ ± 32.3	73.9–197.0	153.4^a^ ± 51.3	85.6–253.3
Ptime (min)	5.6^a^ ± 0.4	3.8–6.3	5.8^a^ ± 0.2	5.5–6.2	5.8^a^ ± 0.3	4.7–6.4	5.7^a^ ± 0.2	5.3–6.2

**Table:** 

**Parameters**	**T1AT (*n* = 6)**		**T1CC (*n* = 6)**		**G2AC (*n* = 2)**		**T2AC (*n* = 8)**		**T1CT (*n* = 1)**
	**Mean ± SD**	**Range**	**Mean ± SD**	**Range**	**Mean ± SD**	**Range**	**Mean ± SD**	**Range**	
AAC (%)	18.2 ± 8.3	8.1–26.6	20.0 ± 2.1	16.0–21.5	2.7 ± 2.0	1.3–4.2	2.0 ± 0.8	1.1–3.0	20.7
HD (g)	41.3 ± 30.9	12.3–77.7	37.1 ± 8.8	26.8–46.1	7.6 ± 0.5	7.2–7.9	7.8 ± 1.2	6.4–9.8	47.4
ADH (g.s)	−59.4 ± 43.3	−101.7 to −13.8	−46.3 ± 8.3	−55.2 to −36.7	−9.1 ± 1.8	−10.4 to −7.8	−10.0 ± 2.7	−16.2 to −7.8	−52.9
COH	0.6 ± 0.0	0.5–0.6	0.6 ± 0.0	0.6–0.6	0.6 ± 0.1	0.6–0.7	0.6 ± 0.1	0.5–0.7	0.6
PV (RVU)	352.5 ± 63.0	226.1–399.1	328.8 ± 21.4	306.4–364.9	263.5 ± 61.2	220.2–306.8	276.2 ± 34.5	221.8–328.3	305.8
HPV (RVU)	198.4 ± 46.1	133.9–256.1	178.0 ± 27.4	144.4–209.6	138.4 ± 12.3	129.8–147.1	156.6 ± 16.4	134.4–188.5	136.8
CPV (RVU)	345.8 ± 90.5	253.4–478.1	335.0 ± 39.4	278.8–382.9	189.3 ± 9.5	182.6–196.0	208.1 ± 24.5	176.0–253.8	269.5
CS (RVU)	147.4 ± 57.2	96.1–222.0	157.0 ± 15.0	134.5–173.3	50.8 ± 2.8	48.8–52.8	51.5 ± 10.8	35.3–65.3	132.8
SB (RVU)	−6.7 ± 97.7	−120.5 to 100.9	6.2 ± 39.1	−44.3 to 76.5	−74.2 ± 51.8	−110.8 to −37.6	−68.2 ± 23.1	−95.0 to −36.8	−36.3
BD (RVU)	154.2 ± 48.4	92.2–217.3	150.8 ± 28.7	96.8–178.8	125.0 ± 48.9	90.4–159.6	119.7 ± 22.4	87.4–142.5	169.0
Ptime (min)	5.8 ± 0.2	5.6–6.0	5.8 ± 0.2	5.5–6.2	3.7 ± 0.1	3.7–3.8	4.0 ± 0.3	3.7–4.5	5.4

1 *G1AC means a G SNP in intron 1, no 23 bp duplication in exon 2, an A SNP in exon 6 and a C SNP in exon 10 of Waxy gene; n: number of accessions*.

*Different letters in the same line indicate significance at P < 0.05; only the first four SNP haplotype groups were compared for mean AAC*.

For parameters HD, ADH and COH of gel texture, the variations of mean values among haplotype groups were highly correlated with that of AAC. The groups having higher mean AAC also had higher mean HD, COH and lower mean ADH (Table 3). However, there was no strong relationship for parameters of pasting viscosity. The G1AT haplotype with highest mean AAC showed highest mean values for all viscosity parameters, but G1AC haplotype which had the second highest mean AAC showed the lowest mean value for PV and HPV, suggesting a significant effect of exon 10 C/T SNP on PV and HPV. On the other hand, the fluctuation of mean values of CPV, CS, and SB followed the trend of AAC. Ptime did not differ significantly among haplotypes.

### The effect of RM190 SSR polymorphism on AAC related traits

**Figure 3:**
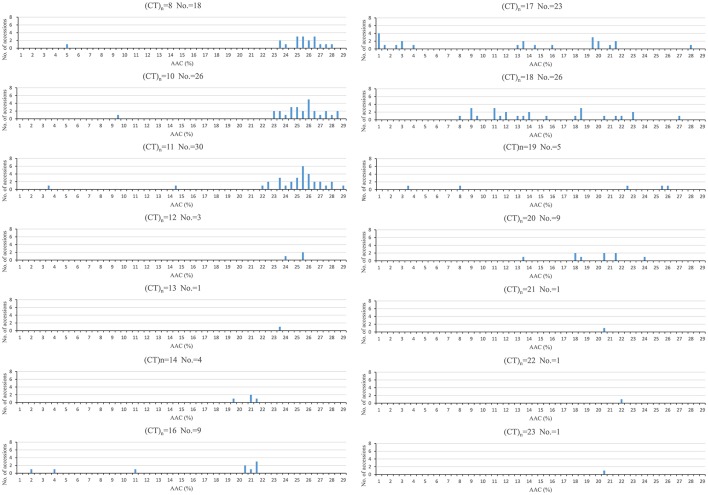
**AAC distribution in association with *Waxy* (CT)_n_ alleles of RM190**. No, number of accessions in each group.

The association between AAC and RM190 polymorphism was visualized in Figure 3. Generally, the shorter repeats of (CT)_8_, (CT)_10_, and (CT)_11_ were primarily associated with high AAC (AAC > 23%), and the range of AAC in these three (CT)_n_ classes was rather narrow. In contrast, the longer (CT)_n_ repeats of 17, 18 covered a wide span of AAC types, primarily ranging from 0 to 23%. For (CT)_14_, (CT)_16_, and (CT)_20_ groups, nearly all accessions had AAC < 23% but the numbers of accessions in these groups were small. The other parameters of AAC-related traits also differed significantly among (CT)_n_ classes (Supplementary Table 4).

Since rice accessions with T1AC and G1AC haplotypes were both fairly evenly distributed in (CT)_8_, (CT)_10_, and (CT)_18_ classes, mean values of AAC-related traits were compared between accessions with same SNP combinations but different (CT)_n_ polymorphisms (Table 4). The results showed that, compared to G1AC haplotype, T1AC haplotype was lower in mean values of AAC, HD, CS, SB, and higher in ADH, PV and BD only in the (CT)_18_ group. In contrast, no traits differed significantly between T1AC and G1AC haplotypes in either (CT)_8_ or (CT)_10_ classes. Furthermore, within T1AC and G1AC, the (CT)_18_ class differed significantly in several parameters when compared with the other two groups. This suggested *Waxy* (CT)_n_ microsatellite polymorphisms had a non-negligible effect on AAC-related traits.

**Table 4:** **Mean and range of AAC related traits in rice accessions with different SNP haplotype and (CT) n combinations**.

**Waxy allele**		**No. of accessions**	**AAC (%)**	**HD (g)**	**ADH (g.s)**	**COH**	**PV (RVU)**	**HPV (RVU)**	**CPV (RVU)**	**CS (RVU)**	**SB (RVU)**	**BD (RVU)**	**Ptime (min)**
**SNPs^1^**	**(CT)_n_**												
T1AC	(CT)_8_	4	26.5^a^	53.9^a^	89.1^c^	0.549^c^	257.7^b^	140.7^b^	316.5^a^	175.8^a^	58.9^a^	117.0^b^	5.53
G1AC	(CT)_8_	13	24.0^ab^	51.8	−81.0^c^	0.570^abcc^	271.2^b^	149.4	320.9^a^	171.6^a^	49.7^a^	121.8^b^	5.51^b^
T1AC	(CT)_10_	6	23.9^ab^	57.7	−85.8^c^	0.552^bc^	292.6^b^	157.7^b^	322.0^a^	164.4^a^	29.5^a^	134.9^b^	5.54^b^
G1AC	(CT)_10_	18	25.9^a^	56.0^a^	−84.0^c^	0.560^abc^	270.4^b^	151.3^b^	335.9^a^	184.6^a^	65.5	119.1^b^	5.58^b^
T1AC	(CT)_18_	12	12.2^c^	17.5^c^	−27.9^a^	0.579^a^	389.6^a^	207.2^a^	327.8	120.6^b^	−61.8^b^	182.4^a^	5.85
G1AC	(CT)_18_	10	18.8^b^	35.5^b^	−53.7^b^	0.572^ab^	311.0^b^	186.0^a^	345.5^a^	159.5^a^	34.5^a^	125.0^b^	5.87^a^

*Different letters in the same column indicate significance at P < 0.05*.

1 *See text for explanation, for example, T1AC means the haplotype with a T SNP in intron 1, no 23 bp duplication in exon 2, an A SNP in exon 6 and a C SNP in exon 10 of Waxy gene*.

### Analysis of variance in AAC related traits

The variances of AAC-related traits explained by each polymorphic site of *Waxy* gene and their combinations (Table 5) showed that for nearly all parameters, the (CT)n microsatellite polymorphism explained the largest portion of the total variance (*R*^2^ = 0.153 − 0.631), followed by the 23 bp InDel of exon 2. The intron 1 G/T SNP alone only explained 16.4% of total variance in AAC (*P* < 0.0001). The exon 6 A/C SNP caused limited variance for all parameters, whereas the exon 10 C/T SNP was greatly responsible for variances in PV, HPV and CPV (*R*^2^ = 0.124 − 0.298; *P* < 0.0001). Interestingly, the SNP combinations explained less variance than RM190 for PV, HPV, SB, BD, HD, ADH, and COH. The three SNP sites together with the 23 bp InDel explained 52.8% of variance in AAC (*P* < 0.0001), but when RM190 was included in the analysis, all the markers together explained as much as 82.0% of the variance (*P* < 0.0001). The effect of RM190 on AAC determination was further highlighted in non-waxy rice (150 accessions) where all the SNPs together only explained 14.6% (*P* < 0.001) of the variance, but RM190 alone could explain up to 48.7% (*P* < 0.0001, Supplementary Table 5). It should be noted, however, the portion of variance of AAC explained by each of the markers and their combinations varied greatly among different sample groups (Supplementary Table 5).

**Table 5:** **Variance of AAC-related traits explained by markers of *Waxy* gene**.

**Parameters**	**RM190**	**Intron 1**	**Exon 2**	**Exon 6**	**Exon 10**	**SNP combinationsa**	**SNPs + RM190**
AAC	0.495^****^	0.164^****^	0.416^****^	0.003	0.044^**^	0.528^****^	0.820^****^
HD	0.611^****^	0.149^****^	0.202^****^	0.034^*^	0.129^****^	0.432^****^	0.783^****^
ADH	0.631^****^	0.115^****^	0.214^****^	0.073^***^	0.073^***^	0.425^****^	0.802^****^
COH	0.218^***^	0.019	0.137^****^	0.004	0.063^**^	0.189^****^	0.561^****^
PV	0.279^****^	0.018	0.037^*^	0.003	0.124^****^	0.266^****^	0.609^****^
HPV	0.436^****^	0.002	0.040^*^	0	0.261^****^	0.375^****^	0.687^****^
CPV	0.455^****^	0.111^****^	0.305^****^	0.004	0.298^****^	0.625^****^	0.792^****^
CS	0.470^****^	0.195^****^	0.398^****^	0.007	0.144^****^	0.612^****^	0.820^****^
SB	0.377^****^	0.190^****^	0.139^****^	0.012	0.054^**^	0.343^****^	0.659^****^
BD	0.153^*^	0.059^**^	0.01	0.009	0.002	0.125^**^	0.446^***^
Ptime	0.232^***^	0.033^*^	0.691^****^	0.029^*^	0.030^*^	0.714^****^	0.851^****^

*^a^SNP combinations include Intron 1, Exon 2, Exon 6 and Exon 10*.

*,**,***,**** indicate significance at P < 0.05, 0.01, 0.001, and 0.0001, respectively

### GT related traits and *SSIIa* alleles in 160 rice accessions grown in hainan

Based on the SNP sites of *SSIIa* gene, three haplotypes were identified. Out of the 160 accessions harvested from Hainan, 124 had the GC-G haplotype (GC SNPs at 4,329–4,330 bp sites and G SNP at 4,198 bp site), indicating this was the most frequent haplotype in this set of accessions. The other two haplotypes had fewer accessions, with 24 (TT-G) and 12 (GC-A) accessions respectively (Table 6).

**Table 6:** **Mean and range of gelatinization temperature related traits in rice accessions with different *SSIIa* alleles**.

	**GCA^1^ (*n* = 123)**		**GCG (*n* = 13)**		**TTG (*n* = 24)**	
**Parameters**	**Mean ± SDc**	**Range**	**Mean ± SD**	**Range**	**Mean ± SD**	**Range**
PT (°C)	73.6b ± 3.2	67.8–78.3	76.8a ± 1.8	67.8–80.8	72.3c ± 3.0	67.6–80.3
To (°C)	63.7c ± 3.9	56.8–71.9	71.7a ± 2.3	61.8–76.6	65.0b ± 2.4	61.0–71.3
Tp (°C)	71.1b ± 2.9	66.7–76.9	76.5a ± 2.0	67.2–81.2	70.9b ± 2.2	67.0–77.0
Tc (°C)	77.0b ± 2.6	72.6–81.4	81.4a ± 2.5	71.8–89.7	77.3b ± 3.8	71.7–91.0
ΔT1/2 (°C)	9.5a ± 2.4	7.2–16.6	6.6c ± 1.7	3.8–14.1	8.2b ± 2.0	4.5–12.9
ΔHg (J/g)	8.4a ± 3.1	4.0–14.9	10.1a ± 7.5	1.8–71.7	8.5a ± 4.9	2.6–27.8
R (%)	5.9^a^ ± 6.5	0.09–20.8	10.3^a^ ± 7.4	0.2–55.0	6.3^a^ ± 6.1	0.08–22.0

*Different letters in the same column indicate significance at P < 0.05*.

1 *See text for explanation, for example, GCA means the haplotype of GC SNPs at 4,329–4,330 bp and an A SNP at 4,198 bp of SSIIa gene. n, number of accessions*.

Gelatinization temperature ranged from 67.2 to 81°C, 67 to 76.7°C, and 66.7 to 73.4°C in haplotypes GC-G, TT-G and GC-A respectively. The GC-G haplotype had significant higher mean PT, T_o_, T_p_, and T_c_ but lower ΔT_1/2_ than the other two haplotypes. The variance explained by the *SSIIa* SNPs ranged from 20.6% to 60.4% for these five parameters (Table 7). On the other hand, ΔHg and R% did not differ significantly among these *SSIIa* haplotypes (Table 6).

**Table 7:** **Variance of gelatinization temperature related traits explained by SNPs in SSIIa gene**.

**Parameters**	**SSIIa GC/TT**	**SSIIa G/A**	**SNPsa**
PT	0.295^****^	0.061^**^	0.396^****^
To	0.298^****^	0.231^****^	0.604^****^
Tp	0.333^****^	0.153^****^	0.551^****^
Tc	0.166^****^	0.097^****^	0.299^****^
ΔT1/2	0.054^**^	0.128^****^	0.206^****^
ΔHg	0.006	0.003	0.010
R	0.029^*^	0.020	0.056^*^

*^a^SNPs include GC/TT SNPs at 4,329–4,330 bp and G/A SNP at 4,198 bp of SSIIa gene*.

*,**,***,**** *indicate significance at P < 0.05, 0.01, 0.001, and 0.0001 respectively*.

## Discussion

Rice is the most important food crop in Asia, and it feeds about half of human population world-wide. Phenotypic variation in natural and cultivated rice and the genetic basis underlying complex traits have been the focus of extensive research. With the advance of genome-wide association analysis, candidate genes or genetic architecture associated with important traits, such as rice grain size and weight, flowering time, disease resistance, abiotic stress tolerance, and metabolites have been the focus of recent studies (e.g., Huang et al., 2010, 2012; Famoso et al., 2011; Zhao et al., 2011; Chen et al., 2014). However, genome-wide association studies to identify genes or gene markers for starch quality traits are relatively few. The USDA rice mini-core collection consists of 217 accessions originated from 76 countries and more than 14 geographical regions worldwide. These diverse accessions are valuable for making new discoveries about genetic determinants of starch quality traits.

### Genetic variation among ancestry groups in the USDA rice mini core collection

As shown in Figure 1, the 26 markers derived from 18 starch biosynthesis related genes could separate the seven ancestry groups in the mini core rice, as classified by Li et al. (2010), into two deeply divided clusters, but they are not sufficiently diverse to separate the ancestry groups within each cluster. This pattern of genetic divergence could provide an explanation for the conflicting findings about rice starch physicochemical properties in some previous studies. For example, some studies reported that AAC was positively correlated with PV whereas others found that the two parameters were negatively correlated or had not a strong relationship (Caffagni et al., 2013; Yang et al., 2014; Kong et al., 2015). This kind of discrepancy is probably due to different sample constitutions in different studies. As shown in the present study (Table 3), due to the effect of exon 10 C/T SNP, high AAC rice could have high or low PV. The relationship between AAC and PV thus could be different depending on the percentage of different *Waxy* gene haplotypes in the germplasm used by different studies. On the other hand, the importance of a marker on certain phenotypic trait could be overestimated due to a narrow genetic base of the samples, as shown in Ayres et al. (1997). Furthermore, the effect of markers from minor genes may be limited to certain populations (Lu and Park, 2012). Taking these factors into consideration, we performed two association analyses on a large number of accessions planted in two environments in order to find the markers with potential to discriminate phenotypic variations in diverse rice germplasm contained in the mini-core collection. The marker-trait associations identified in both analyses were considered to be reliable and strong associations.

### Marker-trait association identified in this study

The genetic basis of rice starch physicochemical properties has been studied previously. Among over twenty starch biosynthesis related genes, *Waxy* gene was reported as the primary gene responsible for amylose content by multiple QTL mapping studies (He et al., 1999; Tan et al., 1999; Septiningsih et al., 2003; Xu et al., 2015). In the previous association analyses, a total of five polymorphic sites in *Waxy* gene were reported as having important effects on AAC variation (Ayres et al., 1997; Larkin and Park, 2003; Wanchana et al., 2003; Chen et al., 2008; Asante et al., 2013; Caffagni et al., 2013). In our association analyses, we included five markers specific to all the five polymorphic sites (G/T SNP of intron 1, 23 bp InDel of exon 2, G/C SNP of exon 6 and C/T SNP of exon 10, and (CT)_n_ repeats) in order to compare their relative importance. We found that the 23 bp InDel in exon 2 and (CT)_n_ microsatellite polymorphisms were most strongly associated with AAC while intron 1, exon 6, and exon 10 were less significant as suggested by the *P*-values (Table 2), and these findings were confirmed by both association analyses.

The *SBEIIb* C/G SNP was reported to be significantly associated with amylose content and viscosity properties (Lu and Park, 2012). However, it should be pointed out that this association was only found for certain populations in the previous study. The present study included diverse rice accessions of different ancestry, but the purported associations involving *SBEIIb* C/G were not found. Similarly, several markers were identified having association with some starch traits, but the associations were not consistently found for both Hainan and Hangzhou samples. This type of inconsistency suggested that compared to *Waxy* and *SSIIa* genes, these markers may have minor or environment-dependent effects on starch traits. For further evaluation of their roles in determining starch characteristics, constructing near-isogenic-lines could be a suitable approach, as demonstrated in recent reports (Luo et al., 2015; Fan et al., 2017).

The *SSIIa* gene was identified as the major gene responsible for GT variation in several QTL mapping studies (He et al., 1999; Tan et al., 1999; Tian et al., 2005; Umemoto and Aoki, 2005). It is not surprising that the association analyses performed in the present study found that the *SSIIa* markers were highly associated with thermal properties, such as that the GC allele of *SSIIa* gene was usually associated with high or intermediate gelatinization temperature (GT) while the TT allele was usually associated with low GT, in agreement with the previous report, e.g., Bao et al. (2006b). The SNP A at the 4198 bp site inactivated the enzyme and thus resulted in low GT regardless of which allele (GC/TT) was present at the 4,229–4,330 bp sites (Nakamura et al., 2005). However, we found that six accessions in the GC-G group had GT lower than 72°C, and three accessions in the TT-G group and one in the GC-A group had GT higher than 72°C. These exceptions indicate that factors influencing GT of rice are complex which warrants further investigations.

In addition, our analysis showed that the *SSIIa* markers were highly associated with AAC-related traits (AAC, HD, etc.). Recently, Fan et al. (2017) also detected the effect of *SSIIa* gene on AAC and viscosity parameters using near-isogenic lines. Thus, the effect of *SSIIa* gene on AAC-related traits could not be purely attributed to gene linkage effect although the *SSIIa* GC SNPs were found associated with the G SNP of intron 1 in *Waxy* gene in most cases in this study (103 out of 137, 75.2% percent).

Previous QTL mapping studies reported that paste viscosity and gel texture were controlled by *Waxy* gene (Bao et al., 2000) or *SSIIa* gene (Wang et al., 2007). The present study showed that for the parameters of AAC-related traits, the highly associated markers were mainly from *Waxy* gene (*P* < 0.001). For these starch parameters, limited markers from other genes could be detected at *P* < 0.01 under *Waxy* background. In contrast, *Waxy* markers prevailed under *SSIIa* background. These findings suggest that *Waxy* gene plays a dominant role in determining AAC-related traits. For GT-related traits, the GC/TT SNPs of *SSIIa* were highly associated with pasting temperature in both Hainan and Hangzhou samples. This association was still significant even under *Waxy* background. Interestingly, although *Waxy* exon 10 C/T SNP was not detected as playing an important role in determining AAC, it was highly associated with HPV and CPV in both Hainan and Hangzhou environments (Table 2). Traore et al. (2011) postulated that exon 10 SNP varied viscosity parameters by causing a proportional change of insoluble to soluble amylose. However, as Hanashiro et al. (2008) reported that *Waxy* gene is also responsible for biosynthesis of the extra-long unit chains of amylopectin in rice which is correlated with viscosity parameters (Han and Hamaker, 2001; Horibata et al., 2004; Inouchi et al., 2005), the putative variation in amylopectin structure between rice with exon 10 C/T SNP should be investigated as another possible cause for variation in viscosity parameters in future studies.

### New *Waxy* haplotypes identified in USDA rice mini-core collection

The 160 rice accessions harvested from Hainan were further analyzed for relationship between *Waxy* haplotypes and variation in AAC related traits. Based on the three SNPs and the 23 bp duplication of *Waxy* gene, we identified a total of nine *Waxy* haplotypes, and two of them (T1CT and T1AT) are reported for the first time. These two new haplotypes were found in seven rice accessions that all had a T SNP in intron 1 of *Waxy* gene, but four of them had AAC > 18%. Previously, the G to T mutation was reported leading to a reduced efficiency of GBSS pre-mRNA processing, which subsequently resulted in a lower level of spliced mature mRNA and lower AAC (Bligh et al., 1998; Cai et al., 1998; Hirano et al., 1998; Isshiki et al., 1998). Ayres et al. (1997) sequenced 42 cultivars and found all the cultivars with amylose content <18% had the T SNP at the intron 1 putative 5′ splice site. Larkin and Park (2003) reported the same results in 14 rice accessions. Chen et al. (2008) measured 53 rice accessions with this T SNP, and nearly all of them showed AAC <18%. However, several studies reported some rice cultivars with the TAC waxy haplotype had AAC > 18% (Dobo et al., 2010; Asante et al., 2013; Caffagni et al., 2013; Biselli et al., 2014). Our present result also showed that the T SNP in intron 1 of *Waxy* gene is not always accompanied by an AAC lower than 18% (Table 3). However, the mechanism of how the SNPs of exon 6 and exon 10 affect GBSS function and starch characteristics remains unclear. It could be due to the amino acid substitutions caused by the two SNPs (Larkin and Park, 2003) that in theory can lead to structural differences affecting GBSS activity and hence starch characteristics. The diverse *Waxy* gene haplotypes identified in the mini-core collection may provide suitable materials for further studies to better understand the structural and functional differences among various GBSS enzymes.

### The (CT)n repeats of *Waxy* gene has a non-negligible effect on AAC related traits

Among the 14 (CT)_*n*_ classes, the most frequent were (CT)_8_, (CT)_10_, and (CT)_18_. The ranges of AAC in rice accessions of the (CT)_8_ and (CT)_10_ classes were quite narrow whereas a wide range of AAC was presented in rice of the (CT)_18_ class. To further examine the effect of (CT)_*n*_ polymorphism on amylose content, we compared rice accessions with the same SNP combinations but different (CT)_n_ repeats. The results showed that T1AC rice had significant lower mean AAC than G1AC rice only in the (CT)_18_ class. Furthermore, when coupled with (CT)_18_, both G1AC and T1AC rice had significantly lower mean AAC than rice with the same SNPs but coupled with (CT)_8_ and (CT)_10_ repeats. This indicated an interaction between SNPs and (CT)_n_ polymorphisms in determining AAC. In addition, our ANOVA results showed that a larger portion of variance of AAC could be explained when RM190 was included in the analysis (Table 5), indicating that RM190 also has a non-negligible effect on AAC. Some previous studies suggested that the SNPs of *Waxy* gene had a superior effect on AAC, hence a priority on SNPs was recommended for utilization in marker-assisted selection (Chen et al., 2008; Dobo et al., 2010; Asante et al., 2013). However, as shown in the present study (Supplementary Table 5), the portion of AAC variance explained by SNPs varied considerably among different ancestry groups, and in some groups, *Waxy* SNPs explained far less variance than RM190. The level of variation in AAC explained by SNPs, RM190 and their combinations varied notably among studies. Using the combination of RM190 and the G/T in intron 1, Ayres et al. (1997) explained 85.9% of the variation in AAC. Dobo et al. (2010) found that 93.8% of the variation in AAC could be explained by the combination of RM190 and the SNPs in intron 1, exon 6 and exon 10. In contrast, both Caffagni et al. (2013) and Biselli et al. (2014) reported a lower level of explained variation with the same markers.

## Conclusion

Using diverse rice accessions in the USDA mini-core collection, we conducted extensive association analyses between 26 molecular markers derived from 18 starch biosynthesis related genes and 18 parameters measured of starch physicochemical properties. We identified many significant marker-trait associations. Furthermore, we investigated marker combinations or haplotype diversity for *Waxy* and *SSIIa* genes in the mini-core rice and their effects on starch traits. In addition to the haplotypes reported in previous studies, we discovered two new haplotypes of *Waxy* gene in seven mini-core accessions. These accessions provided new opportunities for further studies of associations between *Waxy* haplotypes, GBSS enzyme activity and starch quality traits. Compared to *Waxy* SNPs, our analyses showed the (CT)_*n*_ repeat polymorphism also played an important role in determining AAC related traits, thus should not be overlooked in marker-assisted selection (MAS). These findings can help rice breeder develop varieties with improved starch quality via MAS.

## Author contributions

MS initiated the project; KL performed research; JB and HC contributed essential expertise and input in phenotypic and genetic analysis. All authors contributed to the paper writing and approved the final version to be published.

## Funding

This work was supported by grants from General Research Fund of Hong Kong Research Grants Council (Project Code 17106314; 17164716), the National Key Research and Development Program (2016YDF0400104), and the Fundamental Research Funds for the Central Universities (2016XZZX001-09).

### Conflict of interest statement

The authors declare that the research was conducted in the absence of any commercial or financial relationships that could be construed as a potential conflict of interest.
