# Supplementary material for: Association Analysis of Markers Derived from Starch Biosynthesis Related Genes with Starch Physicochemical Properties in the USDA Rice Mini-Core Collection
Source: Front Plant Sci. 2017 Apr 3;8:424. doi: 10.3389/fpls.2017.00424 (PMC5376596; doi:10.3389/fpls.2017.00424)
Supplement: Supplementary file 1 [file SupplementaryTables1-5.docx]

**SUPPLEMENTARY TABLE 1. Mean and range of starch physicochemical properties measured in two environments.**

**SUPPLEMENTARY TABLE 2. Summary of 25 markers derived from starch biosynthesis related genes used in the association analysis.**

**SUPPLEMENTARY TABLE 3. Polymorphism of *Waxy* and *SSIIa* markers in the USDA rice mini-core accessions.^1^**

**Supplementary Table 3** Cont.

**Supplementary Table 3** Cont.

^1^Accession No. = accession number. Accession No. is as designated by Agrama et al. (2010).

**SUPPLEMENTARY TABLE 4. Mean and range of AAC related traits in rice accessions of five (CT)_n_ classes.**

Different letters in the same column indicate significance at *P* < 0.05.

**SUPPLEMENTARY TABLE 5. Variance of AAC explained by *Waxy* RM 190, different SNPs and their combinations in different sample groups.**

^1^ Admix, *accessions with mixed ancestry*; AUS, *aus*; IND, *indica*; JAP, *japonica*.

^2^ SNP combinations include the 23 bp InDel in exon 2.

^*^,^**^,^***^,^****^ indicate significance at *P* < 0.05, 0.01, 0.001, and 0.0001 respectively.
